# Evaluation and quantification of associations between commonly suggested milk biomarkers and the proportion of grassland-based feeds in the diets of dairy cows

**DOI:** 10.1371/journal.pone.0282515

**Published:** 2023-03-02

**Authors:** Amy Birkinshaw, Michael Sutter, Beat Reidy, Laurence Jungo, Stefanie Mueller, Michael Kreuzer, Melissa Terranova

**Affiliations:** 1 ETH Zurich, Institute of Agricultural Sciences, Lindau, Switzerland; 2 Bern University of Applied Sciences (BFH), School for Agricultural, Forest and Food Sciences (HAFL), Zollikofen, Switzerland; 3 Suisselab AG, Zollikofen, Switzerland; 4 ETH Zurich, AgroVet-Strickhof, Lindau, Switzerland; Universidad de Costa Rica, COSTA RICA

## Abstract

This study is a first step approach towards the prediction of the proportion of grassland-based feeds (%GB) in dairy cow diets with the aid of three different groups of milk biomarkers. We aimed to evaluate and quantify the associations between biomarkers commonly suggested in the literature and %GB in individual cows as a hypothesis-generating stage for the prospective establishment of accurate %GB prediction models. Consumers and governments financially encourage sustainable, local milk production making grass-based feeding, in grassland-dominated regions, of major interest. Milk from grassland-fed cows differs from that of other feeding systems by inferential fatty acids (FA), β-carotene content and yellow color; however, these biomarkers have not been evaluated together for their association with %GB. Using approved methods of parametric regression analysis, gas chromatography (GC), mid-infrared spectra (MIR) and color spectroscopy, we aimed to develop a first step towards an easy-to-implement, cost-effective milk-based control to estimate %GB in dairy cow diets. The underlying database was generated with 24 cows each fed one of 24 different diets gradually increasing in grass silage and decreasing in corn silage. Our results indicate that GC-measured α-linolenic acid, total n-3 FA and the n-6:n-3 ratio, MIR-estimated PUFA and milk red-green color index a* are robust milk biomarkers for constructing accurate prediction models to determine %GB. Based on simplified regression analysis, diets containing 75% GB should contain ≥ 0.669 and 0.852 g α-linolenic acid and total n-3 FA per 100 g total FA, respectively, and an n-6:n-3 FA ratio of < 2.02 measured with GC; estimated with MIR, polyunsaturated FA should be ≥ 3.13 g/100 g total FA. β-carotene was not a good predictor for estimating %GB. Unexpectedly, the milk became greener with increasing %GB (negative a* values, ‒6.416 for 75% GB), suggesting the red-green color index, not yellow-blue, as a suitable biomarker.

## Introduction

Consumer awareness and governmental initiative strategies are rapidly expanding when it comes to locally produced, sustainable, climate friendly dairy production [1, 2]. Many governments offer subsidy programs for dairy farmers to encourage sustainable milk production. Feeding grassland-derived forages to dairy cows is traditional and sustainable in countries with temperate climates, abundant rainfall, naturally fertile soil and often, a scarcity of arable land [3]. Concurrently consumers are increasingly willing to pay a premium for dairy products from grass-fed cows that are deemed ethically superior and considered to have a higher nutritional quality than those from corn-silage-concentrate based diets [1]. To benefit from the voluntary grassland-based milk and meat production program (GBMM) in Switzerland, the diet must contain at least 75% grassland-based feed (%GB) and no more than 10% concentrate [4]; for mountain farms the %GB is as high as 85%.

With consumers willing to pay more and farmers being subsidized for grassland-based milk production the need for convenient, cost-effective controls arises. Dairy products from grass-fed cows have higher concentrations of nutritionally beneficial compounds such as vaccenic acid (VA; *trans*-11 C18:1), CLA (conjugated linoleic acids; particularly rumenic acid (RA; *cis*-9, *trans*-11 C18:2)), α- linolenic acid (ALA; C18:3n-3) [3, 5] and β-carotene [6] when compared to corn-silage-concentrate based diets. Mir et al. [7] demonstrated ALA to be the most abundant FA in three of the most commonly fed grasses; orchard grass (*Dactylis glomerata* L.), tall fescue (*Festuca arundinacea* Schreb.) and perennial ryegrass (*Lolium perennne* L). Bär et al. [8] were able to establish a direct relationship between grassland-based feed intake and the FA profile of milk by analyzing the ALA content of bulk milk.

Milk FA are most accurately measured with GC. However, this tedious process requires specialized machinery and multiple, time- and cost-intensive sample preparation steps. Mid-infrared spectroscopy (MIR) is a far quicker measurement routinely used in global milk payment schemes to analyze milk fat, protein, lactose and urea. This convenient method has not been tested for %GB estimation. Another promising indicator for %GB in the diet could be the β-carotene content in milk. This provitamin is a potent natural antioxidant; in both humans and bovines it plays a central role in cell communication and immune function by protecting cells from the scavenging effects of free radicals [9]. Bovines are the only domestic ruminants with the ability to accumulate β-carotene in their milk [10]. The concentration of β-carotene in milk is assumed to be directly proportional to the amount of β-carotene in the cows’ diet [11]. Therefore, β-carotene has repeatedly been suggested as a biomarker in cows’ milk [6, 10–12]. Robust, universal quantification markers require resistance to different influential factors such as the feed storage duration. In this respect, β-carotene is particularly susceptible to degradation via oxidation due to its many double bonds [13]. As the color of dairy products is highly dependent on the concentration of carotenoids such as β-carotene in the milk [6], milk redness and yellowness might be as indicative as β-carotene and offer a much cheaper and quicker way to verify the specifications of grassland-based feeding.

Therefore, the aim of our study was to assess associations between regularly suggested milk biomarkers and relate these back to measured %GB intakes of individual cows as a hypothesis-generating stage to aid future development of robust prediction models using milk biomarkers to predict %GB. We followed a multi-step approach. First, we evaluated the resistance of the suggested milk biomarkers to feed-storage duration. Next, we used an approved parametric regression analysis approach [14, 15] to evaluate the suitability of milk biomarkers to determine grassland-based feeding for a defined %GB and finally we suggested and calculated a preliminary set of the six most promising biomarker prediction values to determine %GB. The following hypotheses were tested. (1) Milk from cows fed diets increasing in %GB has a proportional increase in characteristic FA, β-carotene and red/ yellow milk color. (2) This relationship is dependent on feed-storage duration for β-carotene and color but not for FA.

## Materials and methods

### Animals, diets and experimental design

This experiment was conducted from June to September 2019 in accordance with the ARRIVE guidelines 2.0 [16] and approved by the Cantonal Veterinary Office of Zurich, Switzerland (ZH060/19).

Twenty-four, mid-to-late lactating (321 ± 93 days in milk (DIM); mean ± standard deviation) dairy cows (19 Holsteins and five Brown Swiss) were randomly selected from the research herd at AgroVet-Strickhof (Lindau, Switzerland). Cows were healthy, in 1^st^ to 5^th^ lactation, with an average body weight of 709 ± 80 kg and a milk yield of 17.5 ± 4.0 kg/d. For the purpose of this study each cow comprised a single experimental unit. *A priori* inclusion criteria set were > 100 DIM and a milk yield of < 25 kg/d. To reduce the impact of confounding variables, both groups of 12 cows were matched for breed, lactation number, DIM and average milk yield. Cows were blindly assigned, via opaque envelopes, to one of the 12%GB. This was done in an attempt to quantify biomarker relationships by parametric regression analysis. Variations were accounted for by feeding grass silage and hay harvested in the year of the experiment or a year in advance. We used conserved forage instead of fresh grass as it has a more stable nutrient composition resulting in a more robust regression analysis. Group 1 cows received grass silage and hay harvested in 2018, and Group 2 cows, grass silage and hay from 2019. Exact individual intakes were measured and related back to individual analyzed milk composition. This experiment was conducted in six runs of four cows each. To further minimize confounding factors cows were randomly allocated (drawn from a hat) to runs and each run was randomly allocated either the 2018 or 2019 harvest (2018 harvests were fed in runs 1, 3 and 4, and 2019 harvests in runs 2, 5 and 6).

Grass silage from 2018 was harvested from a mixed (40% *Lolium multiflorum*, Westerwolds ryegrass; 20% *Trifolium incarnatum*; 16.5% *Lolium multiflorum* 2n; 16.5% *Lolium multiflorum* 4n; 7% *Trifolium pratense*), ryegrass-dominated, sward at third and fourth cut at the beginning of ear emergence of the ryegrass. It was ensiled and stored in 700 kg plastic wrapped bales. Grass silage from 2019 was harvested from a ryegrass-clover ley (30% *Lolium perenne*; 30% *Poa pratensis*; 18% *Lolium hybridum*; 12% *Trifolium repens*; 10% *Trifolium arvense*) at third and fourth cut at the beginning of ear emergence of the ryegrass. It was ensiled and stored in a concrete bunker silo. Whole plant corn silage consisted of biomass harvested in autumn 2018 at half milkline stage. It was chopped to theoretical lengths of 10 mm and ensiled and stored in a concrete bunker silo. Warm ventilated hay from both years was harvested at full ear emergence of ryegrass from the second cut of a ryegrass-clover ley.

Twenty-four experimental diets increasing in grass silage proportion and concomitantly decreasing in corn silage proportion were established to form a regression. The intention was to feed the diets with increasing %GB in increments of 5% from 50 to 100%. The diets based on the 2018 harvest, as consumed, consisted of 47, 52, 58, 63, 69, 74, 80, 86, 92, 96, and 100% grassland-based feeds (including 7% hay) on a dry matter (DM) basis, and those based on the 2019 harvest of 52, 57, 62, 67, 73, 78, 85, 89, 93, 96, and 100% (including 8% hay), respectively. The small differences between harvest year groups resulted from variations in DM content of the silages. In each year group the diet containing the lowest %GB (47 and 52%) was fed to two cows, one of which received an additional β-carotene supplement as a β-carotene control. The amount of the β-carotene supplement allocated was 6 g/d for the 2018 group cow, an amount high enough to give a maximum point on the regression line, and 2.8 g/d for the 2019 group cow intended to meet the β-carotene amount ingested with the 100% grassland-based diet from 2019.

A commercial balanced dairy concentrate was supplied at 10% DM to all diets except for the 95% grassland-based diets (5% concentrate) and the 100% grassland-based diet (no concentrate). Realized concentrate proportions as consumed were 7% and 8% for 2018 and 2019, respectively, and 4% each with the two 95% grassland-based diets (realized 96%). These slight differences were due to variations in grass silage DM. The detailed composition of the experimental feeds can be found in S1 Table.

Diets were mixed individually on a daily basis in a TMR mixer. Due to the nature of this feed experiment the primary researcher was aware which cow received which diet. Feed was provided three times per day on individually calibrated built-in floor scales (custom-made model, Mettler-Toledo, Greifensee, Switzerland) and topped up as needed to ensure ad libitum access. Residual feed was weighed and removed every 24 h. Additionally, 150 g/d of a β-carotene free vitamin-mineral mix and 50 g salt were provided. Cows had permanent access to fresh water.

Before the start of a 7-d total collection period, each cow underwent a 10-d adaption period where the new diet was introduced gradually within 5-d from 10% to 100%. Cows were housed individually, side-by-side, in a tied-stall to allow for individual measurements of feed intake. Cows were milked twice daily at 0630 and 1730, milk yield was recorded at each milking. Cows were weighed directly before and after the experimental period and monitored daily for udder and joint inflammation.

### Feed sampling and analysis

Over the course of the experiment, one sample from each of the 2018 grass silage bales used was taken, four were obtained from the 2019 grass silage silo, five from the corn silage silo, and two each from 2018 and 2019 hay and one from concentrate. Grass and corn silage samples were lyophilized, and all feed samples were milled to 1 mm particulate size using a centrifugal mill (model ZM1, Retsch GmbH, Haan, Germany) before analysis. All feed analyses were performed in duplicate.

Contents of DM of feed items were assessed by a thermogravimetric device (TGA-701, Leco St. Joseph, MI, USA), and ether extract (EE) contents were determined using a Soxhlet extractor (B-811, Büchi, Flawil, Switzerland). Fatty acids were extracted with a solvent extractor using a hexane: propane-2-ol mixture (3:2 v/v). They were then transformed to FA methyl esters (FAME) [17] and cleaned following Wettstein et al. [18]. The FAME analysis was performed with a GC equipped with a CP7421 column using split injection (1:5). Internal and external standards applied were C11:0 and commercially available sunflower oil (response factor). The initial temperature was set to 170°C and held for 1 h. This was then increased by 5°C /min to 230°C and held for 32 min, then increased by another 5°C /min to 250°C and held for 15 min. The FA peaks were identified by their retention times using a standard mixture of 37 FA, C4-C24 (Supelco 37-Component FAME Mix CRM47885, Merck, Switzerland) and the Matreya Cis-Trans Isomer standard (MAYA1131; Avantor, Switzerland).

Feed β-carotene content was analyzed with a normal-phase UV/VIS HPLC according to the European Standard method 12823–2 [19].

### Milk sampling and analysis

Milk yield was recorded at each milking (morning and evening, respectively). During the 7-d collection period samples from each milking were pooled in daily aliquots of morning and evening milk proportions. One set of samples was frozen at -20°C, the other was preserved daily with 2-bromo-2-nitro-1,3-propanediol. The latter samples were analyzed for contents of fat, somatic cell count (SCC) and urea with MIR spectroscopy (MilkoScan^™^ FT6000, Foss, Hillerød, Denmark). For FA analysis, the former samples were pooled into one representative sample per cow taking the proportions of each of the 7 days into account when making the pool. Individual milk FA analysis was performed with GC and certain FA (C14:0, C16:0, C18:0, C18:1) and groups of FA (short-chain, medium-chain, long-chain, *trans* FA, SFA, MUFA, PUFA) were determined with MIR by the MilkoScan^™^ 7RM and the new Foss fatty acid origin package. By design the MilkoScan^™^ gives FA results as g/100 g milk. These FA can be automatically converted to g/100 g total FA in the Foss Integrator or by dividing the specific FA (g/100 g milk) by the total fat content (%) × 0.95 × 100 [20]. Milk samples for GC and MIR analyses were defrosted overnight in the refrigerator (4°C). The MIR analysis was conducted the next morning without sample preparation, whereas for GC analysis 0.5 μL of milk was combined with 5 mL of internal standard (n-heptane containing triundecanoin, tetradecenoic methylate and trivaleranoin). Sodium methylate was used for cold trans-esterification to FAME [21]. Response factors were derived from 6:0, 13:0 and 19:0 triglyceride standards. The same GC and column described above for the feeds were used. The FAME were injected at 1.0 μL in a split ratio of 1:1 and 1.7 L H_2_/min. Initially the temperature was set to 60°C and held for 12 min. This was then increased by 5°C /min to 170°C and held for 60 min and then increased by another 5°C /min to 250°C and held for 20 min. FAME identification was performed using a the same Supelco 37-FAME mix and the Matreya Cis-Trans Isomer standard as with the feed items. Individual FA peak identification was confirmed by comparison with chromatograms for milk lipids resulting from similar determinations [22, 23].

Milk β-carotene content was determined using the same method as described above for the feeds. Milk color space (lightness, L*; red-green index, a*; yellow-blue index, b*) was measured in duplicate four times over the 7-day collection period using a Chroma-Meter (Konica Minolta, Ramsey, NJ). For statistical evaluation, these data were averaged to one value per cow.

### Statistical analyses

To aid in the evaluation and quantification of suitable milk biomarkers for %GB a multi-step analysis approach was taken. An initial one-way analysis of variance (using PROC ANOVA of SAS) was performed across both dietary-intake and milk-related variables comparing the years of harvest 2018 and 2019. This allowed the assessment of the stability of potential biomarkers to feed storage duration (year of harvest).

Next, the best model for each response variable was selected based on the lowest corrected Akaike Information Criterion for small samples sizes [24] by stepwise selection of the initial model, depicted as:

$${\text{Y}}_{\text{ij}}={\text{b}}_{0}+{\text{b}}_{1}*\%{\text{GB}}_{\text{ij}}+{\text{b}}_{2}*\%{\text{GBsq}}_{\text{ij}}+{\text{b}}_{3}*{\text{DIM}}_{\text{ij}}+{\text{b}}_{4}*1_{\left\{\text{yearij}=2018\right\}}+{\text{b}}_{5}*\%{\text{GB}}_{\text{ij}}*1_{\left\{\text{year}=2018\right\}}+{\text{e}}_{\text{ij}}$$

where Y = the response variable; i = 1, 2 (for years 2018 and 2019) and j = 1,2, …, n_i_, where n_i_ = 12 (or 11) as equally as many cows received 2018 as 2019 harvests. Let 1_{year_ij = 2018}_ be an indicator function which is 1 if year_ij_ = 2018 and 0 if year_ij_ = 2019; b_0_ = overall mean; b_1_,_2_,…_5_ = regression coefficients of the observed effects of the grass-based diet (%GB, linear and %GBsq, quadratic), of days in milk (DIM) and of year of harvest (l_{year = 2018}_) and e_ij_ = random error. From the initial mode, a stepwise model selection procedure (both forward selection and backward elimination) was applied (using PROC GLMSELECT of SAS). The lower range of the search scope included %GB or %GB squared. Including %GB as both a linear and quadratic term allowed the identification of linear and non-linear (associative) relationships of variables with %GB. To ensure that the dietary effects were corrected for different lactation stages, DIM was included in the initial model as a continuous explanatory variable.

The number of independent datasets (24 for intake data and 22 for milk related traits, the latter excluding the two cows supplemented with β-carotene) approached the threshold given by Jenkins and Quintana-Ascencio [25] for regression analysis with high data variance (≥ 25) and by far exceeded the threshold of ≥ 8 given for low data variance.

**Fig 1:**
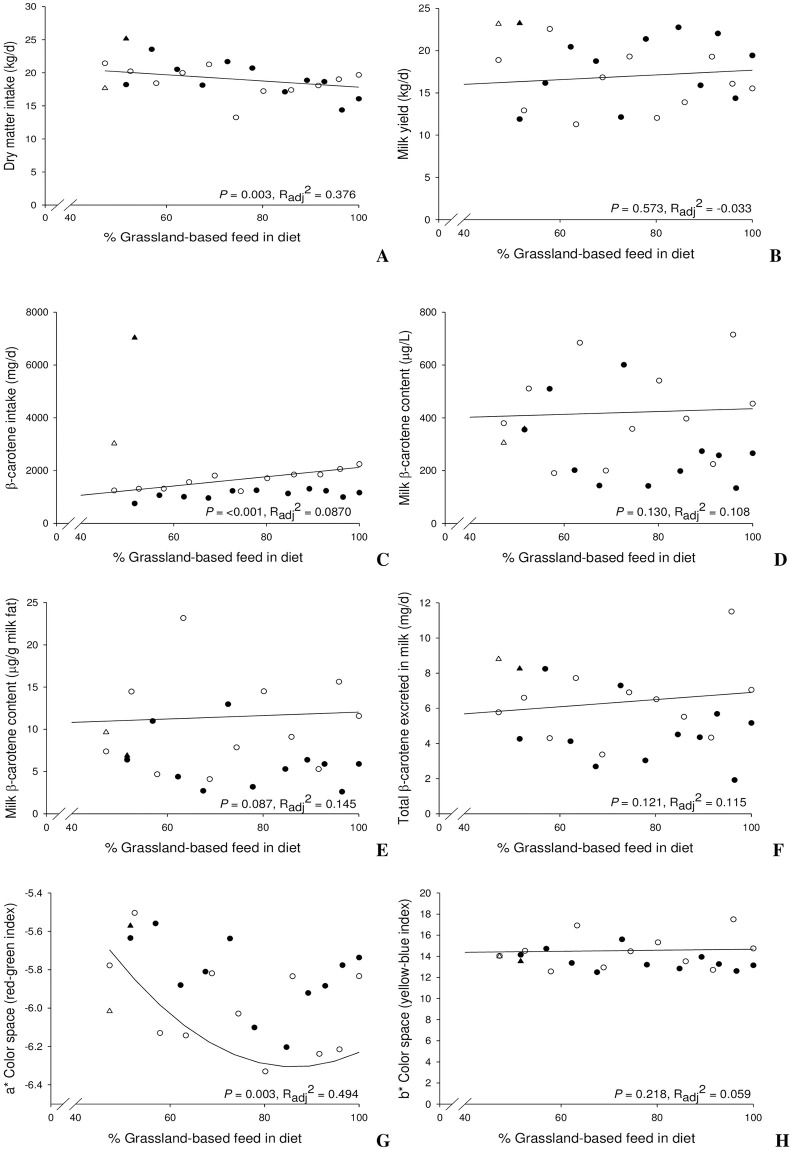
Dry matter intake (A), milk yield (B), β-carotene intake (C), milk β-carotene content in μg/L (D) and μg/g milk fat (E), total daily excretion of β-carotene in milk (F), milk a* color space (G), and milk b* color space (H), in relation to the percentage of grassland-based feed in the dairy cow’s diet. Cows supplemented with β-carotene are depicted as triangles and were excluded from the regressions. Harvest years are presented separately (2018 as filled data points; 2019 as hollow data points). The slope of the regression, *P*-values (*P*) and adjusted R-squared (R_adj_^2^) represent the regression model across all data (for details of regression models see Table 2 and S3 Table.

**Fig 2:**
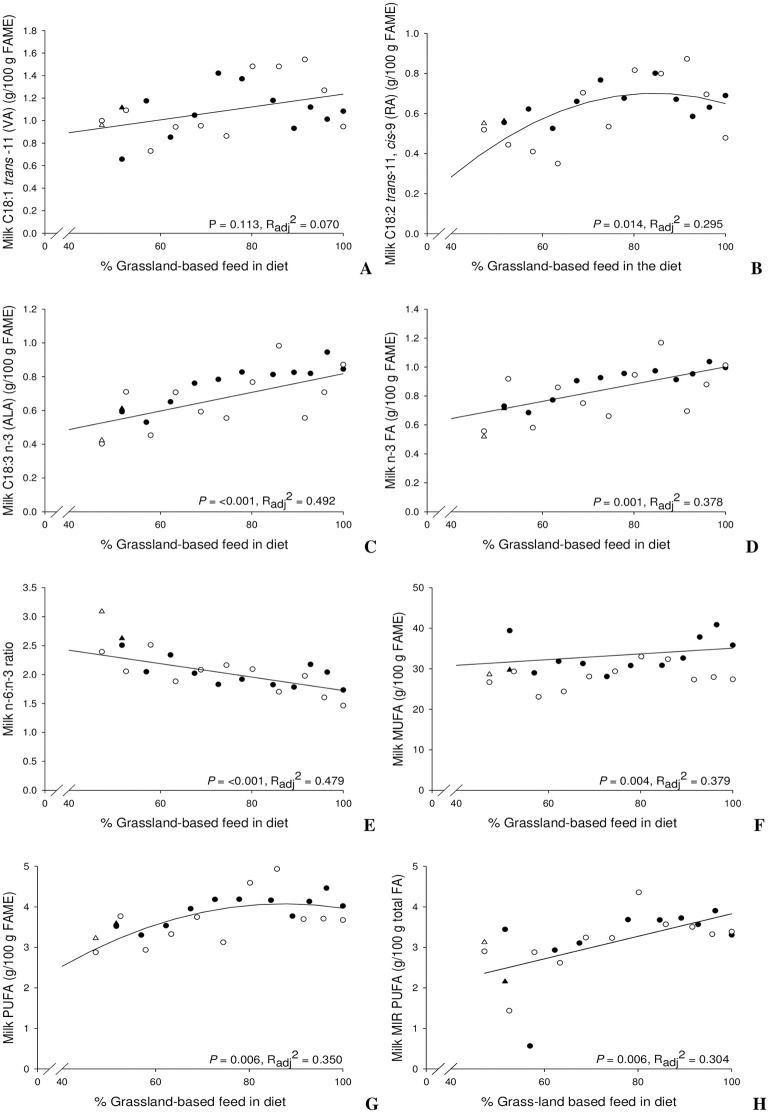
Proportions of C18:1 trans-11 (VA) (A), C18:2 trans-11, cis-9 (RA) (B), C18:3 n-3 (ALA) (C), n-3 (D), n-6:n-3 ratio (E), MUFA (F) and PUFA (G) of total FAME, and MIR-estimated PUFA (H) in relation to the percentage of grassland-based feed in the dairy cow’s diet. Cows supplemented with β-carotene are depicted as triangles and were excluded from the regressions. Harvest years are presented separately (2018 as filled data points; 2019 as hollow data points). The slope of the regression, *P*-values (*P*) and adjusted R-squared (R_adj_^2^) represent the regression model across all data (for details of regression models see Tables 2, 4, 6).

Figs 1 and 2 depict individual cow data. Harvest years are presented separately (2018, filled data points; 2019, hollow data points) and the slope of the regression across all data is depicted as a solid line (the individual data points of the two cows receiving the β-carotene supplement and excluded from the regression analysis are depicted as triangles in the figures). Any time DIM was included in the regression model, it was held constant at the average DIM (321 days). Milk FA regression values are given in Tables 2, 4, 6 and S5 Table.

Lastly, as the first step towards the simplest determination of the six most promising milk biomarker thresholds for a given %GB, DIM and Year were omitted from the model and a simplified regression equation was applied:

$${\text{Y}}_{\text{ij}}={\text{b}}_{0}+{\text{b}}_{1}*\%{\text{GB}}_{\text{ij}}+{\text{b}}_{2}*\%{\text{GBsq}}_{\text{ij}}$$

where b_0_ = the intercept, b_1_ = the coefficient for %GB_ij_ and b_2_ = the coefficient for %GBsq_ij_, based on the previously selected best fit regression model. This allowed for the development of a preliminary set of biomarker prediction values to determine the proportion of dietary grassland-based feeds when the more detailed information of DIM and year of harvest are unavailable. Pearson correlation coefficients were computed with the PROC CORR function of SAS to determine the association between GC and MIR FA data. Effects were considered as statistically significant at *P* < 0.05. All statistical analyses were performed with the statistical software SAS, version 9.4 (SAS Institute, Cary, NC, USA) and figures were generated with SigmaPlot (v. 13; Systat Software, San Jose, CA).

## Results

### Intake and performance

Individual intakes of each cow and intake regression analysis are given in S1–S3 Tables. However, in short, grass silage from 2019 contained 64% more β-carotene than that from 2018, whereas 2018 hay contained 36% more β-carotene than 2019 hay (S2 Table). In all experimental forages ALA was the most abundant FA; proportions were higher by 15% in 2019 grass silage compared to the 2018 harvest. Linoleic acid (LA) was the most abundant FA in concentrate lipids and the second most abundant FA in forage lipids. The proportion of LA, of total FA was highest in corn silage and was higher in hay than in grass silage. Grass silage from 2018 contained 21% more LA than 2019. When comparing classes of FA, polyunsaturated FA (PUFA) made up the highest proportion of FA in the forages, followed by saturated FA (SFA) for grass silage and hay, and monounsaturated FA (MUFA) for corn silage.

Intake of DM and EE declined with %GB, whereas β-carotene intake increased steadily (Fig 1A and 1C and S3 Table). The two cows supplemented with β-carotene had a β-carotene intake intended to be similar to the cow receiving 100% grassland-based feeds in Fig 1C (2019; hollow triangle) or intentionally far higher (7036 mg/d; filled triangle). Regression analysis and analysis of variance revealed that year of harvest was not a significant influential factor on intakes of DM and EE (S3 Table and Table 1). As %GB increased the proportion of certain FA in dietary lipids were affected (S3 Table). Year influenced the proportions of various consumed FA but not LA and VA. With ALA proportion there was a significant interaction with %GB.

**Table 1:** Effects of the year of harvest of grassland-based feeds on intake, ether extract digestibility, milk yield and milk properties.

Item	Year of harvest		Min ‒ Max	*P* value
	2018	2019		
DMI (kg/d)	19.4 ± 3.1	18.6 ± 2.2	13.2 ‒ 25.1	0.484
β-carotene intake (mg/d)^1^	1093 ± 158	1598 ± 378	750 ‒ 2242	<0.001
Ether extract intake (kg/d)	0.70 ± 0.13	0.67 ± 0.08	0.49 ‒ 0.92	0.564
Milk yield (kg/d)	18.2 ± 4.0	16.8 ± 3.9	11.3 ‒ 23.3	0.399
Milk properties				
Fat (%)	4.70 ± 0.51	4.08 ± 0.52	2.95 ‒ 5.60	0.007
Protein (%)	3.70 ± 0.62	3.71 ± 0.37	3.40 ‒ 5.29	0.962
Lactose (%)	4.63 ± 0.19	4.64 ± 0.18	4.36 ‒ 4.88	0.816
SCC (10^3^ cells/ml)	189 ± 114	132 ± 45	80 ‒ 508	0.124
β-carotene (μg/g milk fat)	6.13 ± 3.12	10.61 ± 5.57	2.71 ‒ 23.18	0.024
β-carotene (μg/L)	286 ± 147	413 ± 176	133 ‒ 715	0.069
L* (lightness)	75.5 ± 0.7	76.2 ± 1.5	74.0 ‒ 79.1	0.163
a* (red-green index)	‒5.81 ± 0.20	‒5.99 ± 0.24	‒6.33 ‒ ‒5.50	0.060
b* (yellow-blue index)	13.6 ± 0.9	14.4 ± 1.6	12.5 ‒ 17.5	0.108
Transfer (%) from feed to milk				
β-carotene	0.40 ± 0.19	0.39 ± 0.13	0.12 ‒ 0.78	0.805
C18:3n-3 (ALA)	7.06 ± 1.61	4.82 ± 1.02	3.69 ‒ 9.27	0.001
C18:2 n-6 (LA)	19.2 ± 11.5	13.3 ± 5.4	6.3 ‒ 42.7	0.117
trans-11 C18:1 (VA)^2^	5.76 ± 1.87	4.94 ± 1.49	2.36 ‒ 8.44	0.245
*cis*-9, *trans*-11 C18:2 (RA)^2^	3.45 ±1.36	2.72 ± 1.00	0.97 ‒ 5.73	0.108

Arithmetic means ± SD, Min − Max = overall lowest and highest values (n = 12, 2018; n = 12, 2019), *P* values = one-way ANOVA with Year (categorical) as explanatory variable.

DMI, dry matter intake; SCC, somatic cell count; ALA, α-linolenic acid; GLA, γ-linolenic acid; RA, rumenic acid; VA, vaccenic acid; SD = standard deviation.

^1^Excluding the intakes of the cows supplemented with β-carotene.

^2^In relation to intakes of LA and ALA.

Milk yield was not significantly influenced by %GB (Table 2) and year of harvest (Fig 1B, Table 1). Milk fat content increased with %GB (Table 2) and was lower when feeding the 2019, as opposed to the 2018, harvest (Table 1). Milk protein, lactose and somatic cell count did not differ between harvest years (Table 1). Regression analysis demonstrated a trend for an increase in milk protein with increasing DIM (Table 2).

**Table 2:** Regression coefficients for milk-related traits in relation to the percentage of grassland-based feeds in the diet (%GB) and the year of harvest (n = 11, 2018; n = 11, 2019).

Target variable	Intercept	Explanatory variables						Model	
	b_0_	%GB	%GBsq	Year	%GB × Year_2018_	%GB × Year_2019_	DIM	Adj. R^2^	*P* value
Milk yield (kg/d)	14.9	0.0279	‒^1^	‒	‒	‒	‒	‒0.033	0.573
Milk properties							‒		
Fat (%)	3.41	0.00905	‒	2.35*	‒0.0234	0	‒	0.249	0.043
Protein (%)	3.27	‒0.0412	‒	‒	‒	‒	0.00235*	0.190	0.052
Lactose (%)	4.90	‒0.00349	‒	‒	‒	‒	‒	0.060	0.141
SCC (cells/ml)	50880	1499	‒	‒	‒	‒	‒	0.029	0.217
β-carotene (μg/l)	383	0.538	‒	‒	‒1.96*	0	‒	0.108	0.130
L* (lightness)	78.1	‒0.0229	‒	‒0.863	‒	‒	‒	0.180	0.059
a* (red-green index)	‒3.66	‒0.0690**	0.0004*	‒	0.00183	0	0.00102*	0.494	0.003
b* (yellow-blue index)	14.2	0.00489	‒	‒	‒0.0132	0	‒	0.059	0.218

^1^Variable not included in the final regression model after the variable selection procedure.

***P < 0.001,

**P < 0.01,

*P < 0.05.

### Milk fatty acids

Table 3 depicts the proportion of milk FA, in GC measured total FAME, resistant to year of harvest (S4 Table depicts those affected), this included all short- and medium-chain FA except C8:0. Of the key PUFA, LA proportion was 19% lower when feeding the 2019 harvest compared to 2018. Accordingly, the proportion of total n-6 FA differed between harvest years, as did total SFA and MUFA. There was no difference between years in proportions of ALA, RA, VA, total n-3 FA, and the n-6:n-3 FA and LA:ALA ratios. Feeding 2019 harvests decreased ALA transfer from feed to milk compared to 2018, transfer rates of other key FA did not differ between harvest years (Table 1).

**Table 3:** Individual milk fatty acids or groups of fatty acids (g/100 g of total FAME) proportions unaffected by the year of harvest of grassland-based feeds as analyzed by GC.

Item	Year of harvest		Min ‒ Max	*P* value
	2018	2019		
C8:0	0.09 ± 0.01	0.09 ± 0.01	0.07 ± 0.12	0.163
*iso*-C13:0	0.16 ± 0.04	0.16 ± 0.03	0.11 ‒ 0.24	0.755
*iso*-C14:0	0.28 ± 0.06	0.30 ± 0.04	0.17 ‒ 0.41	0.204
C14:1	1.15 ± 0.26	1.15 ± 0.26	0.77 ‒ 1.65	0.994
C15:0	1.26 ± 0.16	1.37 ± 0.14	1.06 ‒ 1.66	0.107
*iso*-C15:0	0.03 ± 0.01	0.03 ± 0.01	0.02 ‒ 0.05	0.197
C15:1	0.35 ± 0.09	0.31 ± 0.07	0.22 ‒ 0.52	0.295
C16:0	30.6 ± 2.2	30.6 ± 4.3	25.4 ‒ 40.4	0.998
C16:1	0.55 ± 0.05	0.56 ± 0.04	0.46 ‒ 0.66	0.710
C18:0	10.2 ± 1.4	11.1 ± 2.4	7.6 ‒ 16.3	0.259
*cis*-12 C18:1	0.18 ± 0.03	0.17 ± 0.04	0.10 ‒ 0.25	0.524
*trans*-8 C18:1	0.24 ± 0.04	0.26 ± 0.05	0.13 ‒ 0.38	0.198
*trans*-9 C18:1	0.16 ± 0.01	0.18 ± 0.04	0.12 ‒ 0.25	0.179
*trans*-10 C18:1	0.29 ± 0.12	0.28 ± 0.08	0.13 ‒ 0.49	0.940
*trans*-11 C18:1 (VA)	1.08 ± 0.21	1.11 ± 0.27	0.66 ‒ 1.54	0.805
*cis*-9, *cis*-11 C18:2	0.005 ± 0.003	0.007 ± 0.004	0.001‒ 0.015	0.289
*cis*-9, *trans*-11 C18:2 (RA)	0.65 ± 0.08	0.60 ± 0.17	0.35 ‒ 0.87	0.397
*trans*-11,*cis*-15+*trans*-9,*cis*-12 C18:2	0.34 ± 0.10	0.38 ± 0.11	0.21 ‒ 0.59	0.376
*trans*-6 C18:2	0.08 ± 0.02	0.10 ± 0.03	0.05 ‒ 0.16	0.036
*cis*-9,*trans*-13+*trans*-8,*cis*-12 C18:2	0.23 ± 0.07	0.20 ± 0.02	0.16 ‒ 0.38	0.101
*trans*-9, *trans*-11 C18:2*	0.02 ± 0.005	0.02 ± 0.01	0.01 ‒ 0.06	0.438
C18:3n-3 (ALA)	0.75 ± 0.12	0.64 ± 0.18	0.40 ‒ 0.98	0.107
C18:3n-6 (GLA)	0.04 ± 0.004	0.04 ± 0.007	0.03 ‒ 0.05	0.717
C20:0	0.15 ± 0.02	0.15 ± 0.03	0.11 ‒ 0.24	0.767
C20:1n-9	0.05 ± 0.009	0.04 ± 0.009	0.03 ‒ 0.07	0.134
*cis*-11 C20:1	0.17 ± 0.02	0.15 ± 0.03	0.11 ‒ 0.21	0.110
C20:2n-6	0.02 ± 0.007	0.02 ± 0.004	0.01 ‒ 0.04	0.204
C20:3n-6	0.07 ± 0.02	0.07 ± 0.02	0.04 ‒ 0.11	0.643
C20:4n-6	0.10 ± 0.02	0.10 ± 0.02	0.06 ‒ 0.13	0.732
C20:3n-3	0.02 ± 0.004	0.02 ± 0.005	0.008 ‒ 0.03	0.566
C20:4n-3	0.009 ± 0.003	0.009 ± 0.003	0.005 ‒ 0.02	0.792
C20:5n-3 (EPA)	0.04 ± 0.009	0.05 ± 0.01	0.03 ‒ 0.07	0.211
C22:0	0.06 ± 0.01	0.06 ± 0.01	0.04 ‒ 0.09	0.601
C22:1	0.04 ± 0.01	0.05 ± 0.02	0.01 ‒ 0.09	0.329
C22:4n-6	0.04 ± 0.01	0.04 ± 0.01	0.02 ‒ 0.08	0.470
C22:5n-6	0.008 ± 0.002	0.009 ± 0.003	0.004 ‒ 0.01	0.390
Σ PUFA	3.90 ± 0.35	3.64 ± 0.62	2.88 ‒ 4.94	0.207
Σ n-3	0.88 ± 0.12	0.80 ± 0.20	0.52 ‒ 1.17	0.224
n-6:n-3 ratio	2.07 ± 0.29	2.09 ± 0.44	1.46 ‒ 3.09	0.926
LA:ALA ratio	1.81 ± 0.31	1.78 ± 0.46	1.12 ‒ 2.78	0.855

Arithmetic means ± SD, Min − Max = overall lowest and highest values (n = 12, 2018; n = 12, 2019), *P* values = one-way ANOVA with Year (categorical) as explanatory variable. ALA, α-linolenic acid; EPA, eicosapentaenoic acid; GLA, γ-linolenic acid; MUFA, monounsaturated fatty acids; LA, linoleic acid; PUFA, polyunsaturated fatty acids; RA, rumenic acid; SFA, saturated fatty acids; VA, vaccenic acid; SD = standard deviation.

Regression analysis revealed an effect of %GB, %GBsq, Year, DIM or the interaction between %GB and Year in the proportion of most milk fat FA and classes of FA (Table 4; non-significant regression models are shown in S5 Table. The proportion of LA (and that of total n-6; both not depicted in the figures) did not clearly respond to %GB, whereas proportions of VA (Fig 2A), RA (Fig 2B), ALA (Fig 2C), total n-3 FA (Fig 2D) and total PUFA (Fig 2G) clearly increased with increasing %GB. A weaker increase occurred with total MUFA proportion (Fig 2F). The ratio of n-6:n-3 FA decreased with increasing %GB (Fig 2E). Observed values hardly deviated from the regression line for the n-6:n-3 FA ratio, and they were very close for n-3 FA proportion and ALA.

**Table 4:** Significant model regression coefficients for proportions of individual fatty acids (mg/100g total FAME) in milk analyzed by GC in relation to the percentage of grassland-based feeds in the diet (%GB) and the year of harvest (n = 11, 2018; n = 11, 2019).

Target variable	Intercept	Explanatory variables						Model	
	b_0_	%GB	%GBsq	Year	%GB × Year_2018_	%GB × Year_2019_	DIM	Adj. R^2^	*P* value
C4:0	1806	1.72	‒^1^	‒	‒2.41^†^	0	‒1.12*	0.288	0.028
C6:0	1745	0.101	‒	‒346***	‒	‒	‒	0.490	<0.001
C10:0	1035	123^†^	‒0.849^†^	‒942***	‒	‒	‒	0.531	0.001
C10:1	332	‒0.128	‒	‒76.2**	‒	‒	‒	0.217	0.038
C12:0	134	134	‒0.940^†^	‒1126***	‒	‒	‒	0.505	0.001
C12:1	116	‒0.107	‒	‒26.2*	‒	‒	‒	0.230	0.032
C13:0	131	‒0.0217	‒	‒42.2***	‒	‒	‒	0.545	<0.001
C14:0	12770	‒6.42	‒	‒	‒22.4*	0	‒	0.261	0.022
*anteiso*-C14:0	423	0.410	‒	‒	‒1.31*	0	0.419*	0.427	0.004
C15:0	1131	3.51^†^	‒	‒	‒1.80*	0	‒	0.192	0.051
C15:1	350	‒1.85*	‒	‒	‒	‒	0.145*	0.390	0.004
*iso*-C16:0	151	0.860^†^	‒	‒	0.383^†^	0	‒	0.275	0.018
C16:1n-7	1764	0.206	‒	‒	10.2***	0	‒	0.539	<0.001
*anteiso*-C16:0	242	‒	0.0406^†^	‒	‒5.48^†^	‒5.98^†^	‒	0.503	0.001
C17:0	483	1.12	‒	74.9**	‒	‒	‒	0.367	0.005
*iso*-C17:0	20.6	0.940***	‒	‒	‒0.422***	0	‒	0.750	<0.001
C17:1	162	0.921	‒	‒	1.60***	0	‒	0.663	<0.001
*anteiso*-C17:0	86.9	0.00969	‒	15.9*	‒	‒	‒0.0923*	0.340	0.015
C18:1n-9	17415	351	‒	‒	57.2**	0	‒	0.336	0.008
*cis*-10 C18:1	233	0.818	‒	‒	‒1.23***	0	‒	0.365	0.005
*cis*-11 C18:1	175	1.93	‒	‒	2.70***	0	‒	0.515	<0.001
*cis*-12 C18:1	226	‒	‒0.0095***	‒	‒	‒	‒	0.401	<0.001
*cis*-13 C18:1	381	0.475	‒	‒	0.549**	0	‒	0.363	0.005
*cis*-14+*trans*-16 C18:1	339	1.30	‒	‒	‒1.98**	0	‒	0.368	0.005
*trans*-6+7+8 C18:1	‒273	‒	‒0.111	‒	15.5**	15.9**	‒	0.425	0.005
C18:2n-6 (LA)	1097	‒0.341	‒	‒	3.30**	0	‒	0.335	0.008
*trans*-6 C18:2	‒143	‒	‒0.0366^†^	‒	5.80^†^	6.14*	‒	0.374	0.009
*cis*-9, *trans*-13+*trans* -8, *cis*-12 C18:2	123	1.56*	‒	‒	‒0.546*	0	‒	0.252	0.024
*cis*-9, *trans*-11 C18:2 (RA)	‒810	35.8*	‒0.212^†^	‒	‒	‒	‒	0.295	0.014
*cis*-9, *trans*-12 C18:2	‒124	‒	‒0.0358^†^	‒	5.74^†^	6.02*	‒	0.312	0.021
*cis*-9, *cis*-15 C18:2	11.2	0.179	‒	‒	0.184**	0	‒	0.401	0.003
*trans*-11, *cis*-15*+trans*-9, *cis*-12 C18:2	121	3.27*	‒	‒	‒	‒	‒	0.242	0.012
C18:3n-3 (ALA)	255	5.52**	‒	‒	1.07^†^	0	‒	0.492	<0.001
C20:0	224	‒0.962**	‒	‒	‒	0	‒	0.280	0.007
*cis*-5 C20:1	8.23	0.144*	‒	‒	‒0.0744**	0	0.0179^†^	0.341	0.014
*cis*-11 C20:1	76.9	1.04*	‒	99.8*	‒1.08^†^	0	‒	0.256	0.040
C22:6n-3 (DHA)	9.27	0.0373	‒	‒	‒0.0562**	0	‒	0.384	0.004
Σ SFA	73156	‒87.3^†^	‒	‒5308**	‒	‒	‒	0.387	0.004
Σ MUFA	22861	70.4	‒	5182**	‒	‒	‒	0.379	0.004
Σ PUFA	‒1172	120^†^	‒0.686^†^	‒	‒	‒	‒	0.350	0.006
Σ n-3	406	5.95**	‒	‒	‒	‒	‒	0.378	0.001
n-6:n-3 ratio	2885	‒11.6***	‒	‒	‒	‒	‒	0.479	<0.001
LA:ALA ratio	2750	‒ 13.6***	‒	‒	‒	‒	‒	0.561	<0.001

ALA, α-linolenic acid; DHA, docosahexaenoic acid; DIM, days in milk; GB, grassland based; LA, linoleic acid; RA, rumenic acid; VA, vaccenic acid.

***P < 0.001,

**P < 0.01,

*P < 0.05,

^†^*P* < 0.10.

^1^Variable not included in the final regression model after the variable selection procedure.

When testing MIR for milk FA quantification, proportions of most FA and groups of FA determined by Foss software were affected by year of harvest eliminating them as robust biomarkers; however, C16:0, C18:0, *trans* FA and PUFA were not (Table 5). Most MIR-based FA and groups of FA significantly correlated with GC FA results, the simple correlation was closest for C14:0, C18:1, total long-chain FA, total SFA and total MUFA and PUFA had a highly significant %GB effect (Table 6) defining it as the only appropriate MIR measured FA group to ascertain %GB. On closer inspection, the relationship between MIR PUFA and GC PUFA was mostly linear, but also had a non-linear (quadratic) component (see footnote to Table 5).

**Table 5:** Effect of year of harvest of the grassland-based feeds on the proportions of individual fatty acids (FA) or groups of FA (g/100 g of total milk fat) in milk as estimated by MIR.

Item	Year of harvest		Min ‒ Max	*P* value	Correlation^1^
	2018	2019			to GC results
C14:0	8.28 ± 1.41	9.95 ± 0.89	6.14 ‒ 11.87	0.003	0.802***
C16:0	20.87 ± 3.91	23.24 ± 3.67	16.63 ‒ 32.88	0.148	0.245
C18:0	9.37 ± 1.54	8.67 ± 0.88	6.29 ‒ 11.72	0.192	0.362
C18:1	31.06 ± 4.26	26.32 ± 3.07	19.25 ‒ 37.37	0.006	0.852***
Σ Short-chain FA	10.02 ± 0.98	11.17 ± 1.31	7.89 ‒ 12.11	0.038	0.531**
Σ Medium-chain FA	34.71 ± 5.78	39.53 ± 5.19	28.63 ‒ 52.09	0.047	0.476*
Σ Long-chain FA	41.47 ± 5.35	34.35 ± 4.07	26.84 ‒ 49.56	0.002	0.836***
Σ *trans* FA	2.36 ± 0.62	2.88 ± 0.84	1.15 ‒ 4.84	0.110	0.663**
Σ SFA	58.65 ± 4.62	64.33 ± 2.74	51.11 ‒ 68.94	0.002	0.910***
Σ MUFA	30.92 ± 4.20	26.94 ± 2.94	23.38 ‒ 35.97	0.015	0.888***
Σ PUFA	3.10 ± 0.97	3.13 ± 0.69	0.57 ‒ 4.36	0.922	0.492*^2^

Arithmetic means ± SD, Min − Max = overall lowest and highest values (n = 11, 2018; n = 12, 2019), *P* values = one-way ANOVA with Year (categorical) as explanatory variable. MUFA, monounsaturated fatty acids; PUFA, polyunsaturated fatty acids; SFA, saturated fatty acids; SD = standard deviation.

^1^Pearson correlation coefficients, with

*P < 0.05,

**P < 0.01,

***P < 0.001.

^2^Corresponding regression equation: MIR PUFA = 3.054 ‒ 0.736 × GC PUFA + 0.197 × GC PUFA^2^ (RMSE = 0.742, R^2^ = 0.248 *P* = 0.058).

**Table 6:** Regression coefficients for proportions of individual fatty acids (FA) and groups of FA (g/100g total FA) in milk estimated by MIR in relation to the percentage of grassland-based feeds in the diet (%GB) and the year of harvest (n = 10, 2018; n = 11, 2019).

Target variable	Intercept	Explanatory variables						Model	
	b_0_	%GB	%GBsq	Year	%GB × Year_2018_	%GB × Year_2019_	DIM	Adj. R^2^	*P* value
C14:0	12.0	‒0.0284^†^	‒^1^	‒	‒0.0207**	0	‒	0.518	<0.001
C16:0	31.0	‒0.117*	‒	‒	‒	‒	‒	0.208	0.022
C18:0	6.90	0.0278	‒	‒	‒	‒	‒	0.087	0.105
C18:1	18.6	0.104*	‒	4.81*	‒	‒	‒	0.446	0.002
Σ Short-chain FA	‒0.831	0.317^†^	‒0.00198^†^	‒	‒0.0168*	0	‒	0.297	0.029
Σ Medium-chain FA	52.4	‒0.170*	‒	‒4.75*	‒	‒	‒	0.359	0.007
Σ Long-chain FA	24.1	0.134*	‒	7.26**	‒	‒	‒	0.506	0.001
Σ *trans* FA	1.27	0.0217*	‒	‒0.568^†^	‒	‒	‒	0.242	0.032
Σ SFA	71.3	‒0.0933^†^	‒	‒5.73**	‒	‒	‒	0.457	0.002
Σ MUFA	20.3	0.0893^†^	‒	4.14*	‒	‒	‒	0.363	0.007
Σ PUFA	1.05	0.0278**	‒	‒	‒	‒	‒	0.304	0.006

MUFA, monounsaturated fatty acids; PUFA, polyunsaturated fatty acids; SFA, saturated fatty acids.

***P < 0.001,

**P < 0.01,

*P < 0.05,

^†^*P* < 0.10.

^1^Variable not included in the final regression model after the variable selection procedure.

### Milk β-carotene

Effects of %GB were inconclusive for β-carotene content of the milk (Fig 1D and 1E) and β-carotene excretion in milk (Fig 1F); however, the 2019 harvest led to increased β-carotene contents in milk (both per g of milk fat and as a trend (*P* = 0.069) per L of milk; Table 1). The calculated transfer rates of β-carotene from feed to milk did not differ between harvests and were generally low at 0.4%. Supplementation of β-carotene (triangled symbols) did not translate into an increased β-carotene content in milk (μg/L) (Fig 1D and 1E) or daily excretion (mg/day) (Fig 1F).

### Milk color

Across both harvests, a* (red-green color index) was influenced by %GB (milk got greener) and there was a trend (*P* = 0.06) for an effect of the year of harvest (greener for the 2019 harvest) (Fig 1G and 1H, Tables 1 and 2). There was no equivalent relationship for milk L* and b* (yellow-blue color index); b* seemed to increase with %GB in the 2018 harvest and declined with the 2019 harvest (Table 1). The cows that were supplemented with β-carotene (triangled symbols) did not produce milk with a different color compared to the other cows (Fig 1G and 1H).

### Preliminary set of biomarker prediction values to determine the proportion of dietary grassland-based feeds

As a first step towards the simplest determination of the proportion of grassland-based feeds in a dairy cow’s diet, the regression coefficients were omitted for the interaction between %GB and Year in the ALA equation and the interaction between %GB and Year, and DIM, in the a* color equation. This allowed for the development of a preliminary set of biomarker prediction values to determine the proportion of dietary grassland-based feeds when the more detailed information of DIM and year of harvest are unavailable. The following simplified regression equations for milk biomarkers related exclusively to %GB were calculated:

1. GC PUFA (g/100g FAME) = -1.172 + 0.120 × %GB ‒ 0.000686 × %GB^2^ (Root mean square error, RMSE = 0.419, R_adj_^2^ = 0.350, *P* = 0.006);
2. MIR PUFA (g/100 g total FA) = 1.048 + 0.02780 × GB% (RMSE = 0.690, R_adj_^2^ = 0.304, *P* = 0.006)
3. ALA (g/100g FAME) = 0.255 + 0.00552 × %GB (RMSE = 0.110, R_adj_^2^ = 0.492, *P* = <0.001);
4. n-3 FA (g/100g FAME) = 0.406 + 0.00595 × %GB (RMSE = 0.124, R_adj_^2^ = 0.378, *P* = 0.001);
5. n-6: n-3 FA ratio = 2.89 ‒ 0.0116 × %GB (RMSE = 0.199, R_adj_^2^ = 0.479, *P* = <0.001);
6. a* = -3.66 ‒ 0.0690 × %GB + 0.00043 × %GB^2^ (RMSE = 0.169, R_adj_^2^ = 0.494, *P* = 0.003).

This means that for 75%GB (to comply with the Swiss GBMM program^4^ FOAG 2021), 100 g milk fat (FAME) would have to contain at least 3.98 g GC measured PUFA, or 3.13 PUFA as measured with MIR, 0.669 g ALA and 0.852 g n-3. An n-6:n-3 FA ratio of lower than 2.02 and a* of lower than ‒6.416 would be expected for 75%GB. The corresponding values for 85%GB are 4.08, 3.41, 0.724 and 0.912 g/100 g FAME, 1.90 and ‒6.418.

## Discussion

### Fatty acids

Previous studies have demonstrated an increase in key PUFA, particularly n-3 FA, in milk from cows fed grassland-based diets compared to corn-silage-concentrate based diets; due to this, certain key FA have been suggested as indicators for grass and pasture-based feeding regimes [3]. Higher proportions of n-3 FA in milk lipids were repeatedly found in summer versus winter milk, [8, 26, 27] likely because in summer, cows are turned out to pasture and rely less on corn-silage-concentrate based diets [3, 22]. This increase in n-3 FA and other key FA has been reported in the milk of individual cows [5] and in bulk or tank milk [26, 27]. However, few studies have quantified the impact of increasing %GB and the resulting n-3 FA proportions in the milk fat. Bär et al. [8] demonstrated that for every 10% increase in dietary herbage proportion (defined in their study as fresh grass fed on pasture or indoor, grass silage and hay) an increase in proportions of n-3 FA (0.08 g/100 g), CLA (0.08 g/100 g), VA (0.19 g/100 g) and branched-chain FA occurred (GC analyzed bulk milk). Results from our simplified regression equations confirm an increase of n-3 FA, RA (the major CLA) and VA in the milk FA of 0.06, 0.34 and 0.06 g/100 g per 10% additional grassland-based feeds in the diets consumed by individual cows. Very similar to Bär et al. [8] for n-3 but higher for RA and lower for VA in individual cows compared to the reported bulk milk values. Additionally, our results demonstrate that ALA and the n-6:n-3 ratio clearly varied according to %GB and (including total n-3 FA and MIR measured PUFA) were not affected by year of harvest. The corresponding increase of ALA proportions and decrease of the n-6:n-3 FA ratio per 10% of additional grassland based feeds was 0.06 g/100 g and 0.12. Linoleic acid proportion was found to be a significant indicator of %GB in milk as well; however, our results showed a significant difference between harvest year groups and a different slope of response (increase with 2018 harvest, decrease with 2019 harvest) suggesting that this key FA would not be a good predictor of %GB in farm practice. Proportions of RA and VA, not affected by harvest year, varied quite widely from the regression line; RA turned out to be a significant predictor of %GB while VA exhibited a trend.

Subjecting MIR-determined FA proportions in milk fat to regression analysis revealed C16:0, medium-chain, long-chain and *trans* FA as significant predictors of %GB; however, most were affected by year of harvest eliminating them as robust biomarkers. PUFA were not affected by harvest year and were the only group of MIR measured FA highly significant for %GB in regression analysis and subsequent %GB ascertainment. This defines PUFA as measured with MIR as a possible convenient control for %GB ascertainment on farm. However, limited correlation and larger variation of MIR-based PUFA compared to GC-based PUFA data as well as the different simplified regression slopes may limit this approach in individual animals.

### β-carotene

Our results revealed a rather inefficient transfer rate of β-carotene from feed to milk, ≤ 0.4%. Nevertheless, the average of 8.4 μg/g milk fat was about twice as high as those reported by Calderón et al. [28] for rotationally (4.7 μg/g) and strip grazing cows (5.0 μg/g). Our values were very similar to the spring values of 8.3 to 11.0 μg/g reported by Marino et al., [29] who found lower amounts in summer and autumn with 2.1 to 2.8 μg/g and 0.5 to 2.3 μg/g, respectively. There is a high rate of individual variability in milk β-carotene concentrations. Jensen et al. [30] reported differences in milk β-carotene content according to sire and Nozière et al. [6] reported a 46% coefficient of variation in milk β-carotene content among 36 dairy cows receiving a grass silage- or hay-based diet. Additionally, Kuczyńska et al. [31] showed an inverse relationship between milk β-carotene content and SCC. We were unable to prove this inverse relationship in our data (r = 0.186, *P* = 0.385). Six cows in the present study had a SCC > 200 000 indicating probable sub-clinical mastitis with bacterial infection [32]; however, only one cow had a SCC > 400 000 making the majority of the raw milk analyzed in the present experiment compliant with the European Union, EU Council Directive 92/46/EEC [33] on SCC and fit for market consumption within the EU, Switzerland, Norway, Australia and New Zealand.

Over the last two decades β-carotene has consistently been suggested as a potential biomarker for milk from grass-fed dairy cows [6, 10–12]. Consistent with this, the higher β-carotene of the 2019 grass silage clearly promoted β-carotene content in milk and milk fat when compared to feeding the 2018 harvest (where about 1/3 of the β-carotene seems to have been degraded when compared to the 2019 harvest) and thus resulted in a similar transfer efficiency. However, %GB did not translate to an increased β-carotene content and daily excretion in the present study, although β-carotene intakes increased linearly with increasing %GB. Rather surprisingly, none of the supplemented β-carotene was recovered in the milk. We have no obvious explanation for this phenomenon and cannot rule out the possibility that mechanisms triggering β-carotene transformation, during digestion and metabolism, may be differently affected by %GB and β-carotene supplementation rather than by fresh vs. stored forage. *In vitro* and *in vivo* studies considering the role of ruminal digestion on β-carotene provide contrasting results. Dawson and Hemington [34] found no degradation of β-carotene by rumen microbes in sheep, whereas King et al. [35] reported up to 55% loss when β-carotene was incubated with rumen fluid *in vitro*. More recently Yang et al. [36] detected no β-carotene in the serum or body fat of sheep or goats, but it was the carotenoid most present in the serum, fat and liver of cattle. In sheep, all absorbed β-carotene is converted by the gut enterocytes to retinol [37]. In bovines this conversion is less stringent allowing for the accumulation of β-carotene in meat and milk [6].

Due to the high degree of variability in milk β-carotene content and the lack of inverse relationship with SCC, further clarification is required to consider β-carotene in milk as a reliable biomarker for the %GB in dairy cow diets. Although it may be possible to differentiate grassland-based feeding from corn-silage-concentrate based feeding [6, 11] our results suggest that it is not reliably suitable to quantify %GB.

### Milk color

Milk color is determined by a combination of different factors including casein micelles, fat globules, calcium and phosphorus (L*, lightness), and natural pigments including riboflavin (influencing the green index of a*) and carotenoids (influencing the yellow index of b*) [6]. In the present study the milk lightness (L*) was not affected between year groups; however, milk from cows consuming the 2019 harvest depicted a trend towards more yellow milk (+b*). This effect is likely based on the higher carotenoid content of the 2019 feed transferred to milk. In contrast, we did not observe an increase in β-carotene content and consequently, in yellow color with increasing %GB.

However, unexpectedly we did observe a shift towards the–a* color space of the milk with increasing %GB. This means that the higher the %GB, the greener the milk became (not visible to the naked eye). Two greenish-colored compounds offer a plausible explanation for this observation: riboflavin (yellow-green fluorescent hue) and chlorophyll metabolites. Hand and Sharp [38] found that commercial milk in summer contained 20% more riboflavin than in winter. They attributed this to a change in diet from corn silage in winter to pasture in summer. More recently, Laverroux et al. [39] confirmed the increased proportion of riboflavin in tank milk from cows fed grassland-based diets compared to those fed corn-based diets. Another explanation for the green color of our milk samples could be pigments like chlorophyll metabolites, including pheophorbide *a*. Bhattacharjee et al. [40] demonstrated that commercial milk from grass-fed cows was richer in chlorophyll metabolites than milk from grain/silage-fed cows.

## Conclusions

Convenient cost-effective controls are required to differentiate grassland-based milk production from other production systems. Results from the present study provide a first step towards the development of robust prediction models for the determination of the %GB in a cow’s diet from specified milk biomarkers. This should be proceeded by the establishment of large data sets including both individual and bulk milk samples from different countries to construct accurate prediction models. Based on our results, total n-3 FA and the n-3: n-6 FA ratio in milk as measured with GC would be the most consistent, reliable FA predictors of %GB. However, as FA measurement with GC is time consuming and requires trained staff to prepare samples for multi-step analysis, milk PUFA determined with MIR could be a convenient, cost-effective alternative to ascertain %GB in dairy cow diets. GC-measured ALA, n-3 FA, the n-3:n-6 FA ratio and MIR measured PUFA were found to be resistant to feed storage duration and threshold values for a particular %GB can be calculated from our simplified regression equations. Unexpectedly, milk color became greener with increasing proportions of grassland-based feed in the diet. Based on these findings MIR measured PUFA combined with a* color space measurements may be the most cost effective, easy-to-implement control during routine milk analysis for the assessment of the proportion of grassland-based feed in a dairy cow’s diet. To ensure precision measurements, FA data should be periodically validated by GC laboratory analysis of individual FA and FA classes in bulk milk. The above biomarkers require additional research to determine if they are also resistant against other potential factors of influence including the process and quality of feed conservation, vegetation stage at harvest and the replacement of corn silage with fresh grass or grass hay, instead of grass silage.

## Supporting information

S1 Table Dietary intake, composition and analyzed chemical composition of individual experimental diets. (DOCX)

S2 Table Contents of β-carotene, ether extract and fatty acid composition of experimental feeds. (DOCX)

S3 Table Regression coefficients for intake of dry matter, β-carotene, ether extract and proportions of individual fatty acids of total consumed dietary lipids (average of 7 days) in relation to the percentage of grassland-based feed in the diet (%GB) and the year of harvest (n = 12, 2018; n = 12, 2019). (DOCX)

S4 Table Individual milk fatty acids or groups of fatty acids (% of total FAME) proportions affected by the year of harvest of grassland-based feeds as analyzed by GC. (DOCX)

S5 Table Insignificant model regression coefficients for proportions of individual fatty acids (mg/100g total FAME) in milk analyzed by GC in relation to the percentage of grassland-based feeds in the diet (%GB) and the year of harvest (n = 11, 2018; n = 11, 2019). (DOCX)

## Abbreviations

%GB: proportion of grassland-based feeds in diet
ALA: α-linolenic acid
CLA: conjugated linoleic acid
DIM: days in milk
DM: dry matter
EE: ether extract
FA: fatty acid
FAME: fatty acid methyl esters
GBMM: grassland-based meat and milk production
GC: gas chromatography
MIR: mid-infrared spectroscopy
MUFA: monounsaturated fatty acid
PUFA: polyunsaturated FA
RA: rumenic acid
SCC: somatic cell count
SFA: saturated fatty acid
VA: vaccenic acid
