# Supplementary material for: Evaluation and quantification of associations between commonly suggested milk biomarkers and the proportion of grassland-based feeds in the diets of dairy cows
Source: PLoS One. 2023 Mar 2;18(3):e0282515. doi: 10.1371/journal.pone.0282515 (PMC9980782; doi:10.1371/journal.pone.0282515)
Supplement: S1 Table — (DOCX) [file pone.0282515.s001.docx]

**S1 Table. Dietary intake, composition and analyzed chemical composition of individual experimental diets.**

| **Harvest year** | **2018** | | | | | | | | | | | |  | **2019** | | | | | | | | | | | |
| --- | --- | --- | --- | --- | --- | --- | --- | --- | --- | --- | --- | --- | --- | --- | --- | --- | --- | --- | --- | --- | --- | --- | --- | --- | --- |
| **Diet** | **1** | **2** | **3** | **4** | **5** | **6** | **7** | **8** | **9** | **10** | **11** | **12** |  | **13** | **14** | **15** | **16** | **17** | **18** | **19** | **20** | **21** | **22** | **23** | **24** |
| DMI kg/d  g/kg dietary DM | 18.2 | 25.1 | 23.5 | 20.5 | 18.1 | 21.7 | 20.7 | 17.1 | 18.9 | 18.7 | 14.4 | 16.1 |  | 21.4 | 17.7 | 20.2 | 18.4 | 19.9 | 21.3 | 13.2 | 17.2 | 17.4 | 18.1 | 19.0 | 19.7 |
| GB | 516 | 516 | 570 | 622 | 675 | 727 | 778 | 846 | 892 | 928 | 965 | 1000 |  | 472 | 472 | 525 | 579 | 633 | 689 | 745 | 802 | 859 | 916 | 959 | 1000 |
| Grass silage | 445 | 445 | 499 | 552 | 605 | 657 | 709 | 784 | 830 | 785 | 825 | 862 |  | 395 | 395 | 447 | 500 | 554 | 609 | 664 | 721 | 778 | 748 | 793 | 836 |
| Hay | 71 | 71 | 71 | 70 | 70 | 70 | 70 | 63 | 62 | 143 | 140 | 138 |  | 77 | 77 | 78 | 79 | 79 | 80 | 80 | 81 | 81 | 168 | 166 | 164 |
| Corn silage | 413 | 413 | 360 | 307 | 255 | 203 | 152 | 91 | 45 | 0 | 0 | 0 |  | 451 | 451 | 397 | 343 | 288 | 232 | 175 | 118 | 59 | 0 | 0 | 0 |
| Concentrate | 71 | 71 | 71 | 70 | 70 | 70 | 70 | 63 | 62 | 72 | 3.5 | 0 |  | 77 | 77 | 78 | 79 | 79 | 80 | 80 | 81 | 81 | 84 | 41 | 0 |
| Diet composition, g/kg DM as consumed by the individual cow | | | | | | | | |  |  |  |  |  |  |  |  |  |  |  |  |  |  |  |  |  |
| Organic matter | 896 | 896 | 903 | 900 | 898 | 895 | 893 | 880 | 887 | 891 | 889 | 887 |  | 923 | 923 | 928 | 915 | 911 | 907 | 904 | 899 | 895 | 895 | 893 | 891 |
| Crude protein | 147 | 147 | 154 | 159 | 164 | 171 | 176 | 181 | 187 | 184 | 182 | 180 |  | 144 | 144 | 152 | 156 | 161 | 168 | 173 | 180 | 186 | 182 | 180 | 179 |
| Ether extract | 40.5 | 40.5 | 41.0 | 41.0 | 41.1 | 41.2 | 41.3 | 40.7 | 41.1 | 40.1 | 38.4 | 36.2 |  | 41.7 | 41.7 | 42.2 | 42.0 | 42.2 | 42.4 | 42.6 | 42.8 | 43.0 | 40.9 | 38.8 | 36.9 |
| NDF | 495 | 495 | 500 | 499 | 498 | 497 | 497 | 491 | 495 | 506 | 510 | 516 |  | 489 | 489 | 490 | 482 | 480 | 476 | 473 | 470 | 466 | 486 | 491 | 492 |
| ADF | 295 | 295 | 298 | 298 | 298 | 299 | 299 | 296 | 299 | 310 | 313 | 317 |  | 302 | 302 | 306 | 303 | 303 | 304 | 304 | 304 | 305 | 321 | 326 | 329 |
| ADL | 60.8 | 60.8 | 62.9 | 64.2 | 65.6 | 67.2 | 68.6 | 69.9 | 71.7 | 71.1 | 71.8 | 72.7 |  | 33.4 | 33.4 | 33.0 | 32.1 | 31.5 | 30.8 | 30.2 | 29.5 | 28.8 | 30.8 | 29.7 | 28.2 |
| GE (MJ/kg DM) | 18.5 | 18.5 | 18.6 | 18.6 | 18.6 | 18.6 | 18.5 | 18.4 | 18.5 | 18.5 | 18.5 | 18.4 |  | 18.8 | 18.8 | 19.0 | 18.8 | 18.8 | 18.8 | 18.8 | 18.8 | 18.8 | 18.8 | 18.7 | 18.7 |

DMI, dry matter intake; DM, dry matter; GB, proportion of grass-based feeds (grass silage DM + hay DM); NDF, neutral detergent fiber, assayed with α-amylase and corrected for ash concentration; ADF, acid detergent fiber corrected for ash concentration, GE, gross energy.

^1^Cow supplemented with 6.0 g β-carotene per day.

^2^Cow supplemented with 2.8 g β-carotene per day.
