# Supplementary material for: Evaluation and quantification of associations between commonly suggested milk biomarkers and the proportion of grassland-based feeds in the diets of dairy cows
Source: PLoS One. 2023 Mar 2;18(3):e0282515. doi: 10.1371/journal.pone.0282515 (PMC9980782; doi:10.1371/journal.pone.0282515)
Supplement: S2 Table — (DOCX) [file pone.0282515.s002.docx]

**S2 Table**. **Contents of β-carotene, ether extract and fatty acid composition of experimental feeds.**

|  | **Grass silage** | | **Grass hay** | | **Corn silage** | **Concentrate** |
| --- | --- | --- | --- | --- | --- | --- |
| **Year of harvest** | **2018** | **2019** | **2018** | **2019** | **2018** |  |
| Per kg of DM |  |  |  |  |  |  |
| β-carotene (mg) | 82.3 ± 21.2 | 135.3 ± 15.1 | 9.4 ± 2.8 | 6.9 ± 2.8 | 9.3 ± 10.0 | 0.7 |
| Ether extract (g) | 3.45 ± 0.45 | 3.49 ± 0.45 | 1.64 ± 0.28 | 1.40 ± 0.03 | 3.35 ± 0.67 | 8.58 |
| Fatty acids (g/kg total FAME) | | | | | | |
| C8:0 | 0.29 ± 0.16 | 0.18 ± 0.02 | 1.05 ± 0.17 | 1.17 ± 0.27 | 1.87 ± 0.05 | 0.08 |
| C10:0 | 0.34 ± 0.06 | 0.25 ± 0.03 | 1.75 ± 0.18 | 1.84 ± 0.55 | 0.26 ± 0.04 | 0.10 |
| C12:0 | 4.01 ±0.57 | 3.45 ± 0.48 | 35.2 ± 11.0 | 45.9 ± 24.4 | 4.34 ± 0.47 | 0.33 |
| C12:1 | 2.49 ±0.68 | 1.94 ± 0.78 | 2.15 ± 0.06 | 1.74 ± 0.53 | 0.30 ± 0.18 | 0.06 |
| C14:0 | 4.70 ± 0.62 | 3.59 ± 0.25 | 17.7 ± 0.21 | 20.2 ± 0.51 | 2.44 ± 0.20 | 1.02 |
| C15:0 | 1.69 ± 0.28 | 0.89 ± 0.10 | 2.71 ± 0.27 | 2.76 ± 0.11 | 0.39 ± 0.03 | 0.52 |
| C16:0 | 173 ± 11 | 136 ± 0 | 228 ± 17 | 222 ± 21 | 127 ± 9 | 101.0 |
| *iso*-C16:0 | 19.8 ± 0.9 | 18.5 ± 0.9 | 13.3 ± 0.5 | 9.7 ± 0.3 | 13.8 ± 0.4 | 0.6 |
| C16:1 n-7 | 1.96 ± 0.23 | 1.98 ± 0.56 | 4.79 ± 0.27 | 6.17 ± 1.29 | 1.65 ± 0.27 | 2.27 |
| *anteiso*-C16:0 | ‒ | ‒ | ‒ | ‒ | ‒ | 0.25 |
| C17:0 | 1.96 ± 0.16 | 0.97 ± 0.04 | 3.79 ± 0.32 | 3.22 ± 0.12 | 1.36 ± 0.13 | 0.82 |
| C18:0 | 17.5± 1.6 | 10.6 ± 0.6 | 29.0 ± 0.8 | 34.5 ± 206.8 | 26.7 ± 201.7 | 24.9 |
| *trans*-9 C18:1 | ‒ | ‒ | ‒ | ‒ | ‒ | 0.73 |
| *trans*-10 C18:1 | ‒ | ‒ | ‒ | ‒ | ‒ | 0.86 |
| *trans*-11 C18:1 | ‒ | ‒ | ‒ | ‒ | ‒ | 0.76 |
| *trans*-12 C18:1 | ‒ | ‒ | ‒ | ‒ | ‒ | 0.17 |
| *cis*-9 C18:1 | 24.1 ± 3.8 | 22.6 ± 5.1 | 52.4 ± 5.0 | 84.6 ± 7.6 | 314.3 ± 43.5 | 363.1 |
| *cis*-11 C18:1 | 4.4 ± 0.5 | 3.7± 0.5 | 5.8 ± 0.6 | 8.7 ± 2.2 | 5.7 ± 0.8 | 20.3 |
| *cis*-13 C18:1 | ‒ | ‒ | ‒ | ‒ | ‒ | 0.24 |
| C18:2 n-6 (LA) | 148 ± 7 | 122 ± 10 | 173 ± 4 | 175 ± 16 | 456± 5 | 419 |
| C18:3n-6 (GLA) | 1.79 ± 0.63 | 1.93 ± 0.39 | 4.54 ± 0.30 | 5.72 ± 0.41 | 0.48 ± 0.19 | 0.16 |
| C18:3n-3 (ALA) | 560 ± 25 | 645 ± 12 | 342 ± 3 | 295 ± 43 | 35 ± 16 | 43 |
| C20:0 | 6.11 ± 1.55 | 3.74 ± 0.67 | 20.26 ± 0.66 | 20.81 ± 2.02 | 6.55 ± 0.42 | 4.21 |
| C20:1n-9 | 2.10 ± 0.25 | 6.47 ± 1.75 | 3.17 ± 0.01 | 3.24 ± 0.23 | 2.84 ± 0.47 | 5.59 |
| C20:1n-7 | 0.46 ± 0.17 | 0.39 ± 0.09 | 1.71 ± 0.12 | 1.14 ± 0.20 | 0.39 ± 0.31 | 0.19 |
| C21:0 | 0.81 ± 0.10 | 0.39 ± 0.04 | 2.35 ± 0.06 | 2.33 ± 0.26 | 0.56 ± 0.19 | 0.41 |
| C20:2n-6 | 0.88 ± 0.14 | 0.68 ± 0.06 | 1.15 ± 0.11 | 1.18 ± 0.11 | 0.31 ± 0.03 | 0.55 |
| C22:0 | 9.72 ± 0.92 | 6.71 ± 0.02 | 23.7 ± 1.26 | 22.3 ± 2.10 | 3.62 ± 0.26 | 3.94 |
| C20:3n-3 | 0.24 ± 0.09 | 0.12 ± 0.10 | ‒ | ‒ | 0.05 ± 0.03 | 0.02 |
| C22:1 | 1.20 ± 0.22 | 0.78 ± 0.15 | 2.66 ± 0.27 | 2.79 ± 0.14 | 0.34 ± 0.03 | 0.61 |
| C20:5n-3 (EPA) | 0.61 ± 0.19 | 0.29 ± 0.13 | 1.40 ± 0.02 | 1.17 ± 0.13 | 0.34 ± 0.14 | 0.13 |
| C23:0 | 2.31± 0.34 | 0.84 ± 0.05 | 5.43 ± 0.80 | 4.44 ± 0.15 | 1.21 ± 0.10 | 0.54 |
| C22:2 | 0.99 ± 0.23 | 0.39 ± 0.07 | 1.97 ± 0.48 | 3.07 ± 0.03 | 0.61 ± 0.19 | 1.10 |
| C24:0 | 7.77 ± 0.69 | 5.07 ± 0.34 | 18.2 ± 1.26 | 17.08 ± 1.52 | 4.94 ± 0.35 | 2.39 |
| C24:1n-9 | 0.43 ± 0.11 | 0.72 ± 0.04 | 1.16 ± 0.24 | 1.23 ± 0.02 | 0.25 ± 0.23 | 0.59 |
| Σ SFA | 249 ± 15 | 191 ± 1 | 402 ± 3 | 409 ± 51 | 181 ± 9 | 141 |
| Σ MUFA | 37± 4 | 39 ± 3 | 74 ± 5 | 109 ± 8 | 326 ± 43 | 396 |
| Σ PUFA | 713± 19 | 771 ± 3 | 524 ± 2 | 482 ± 58 | 493± 37 | 463 |

Means ± SD, grass silage 2018, n = 9; grass silage 2019, n = 4; hay 2018, n = 2, hay 2019, n = 2; corn silage, n = 5; concentrate, n = 1. ALA, α-Linolenic acid; DM, dry matter; EPA, eicosapentaenoic acid; FAME, fatty acid methyl ester; GLA, γ-linolenic acid; LA, linoleic acid; MUFA, monounsaturated fatty acids; PUFA, polyunsaturated fatty acids; SD, standard deviation; SFA, saturated fatty acids.
