# Supplementary material for: Evaluation and quantification of associations between commonly suggested milk biomarkers and the proportion of grassland-based feeds in the diets of dairy cows
Source: PLoS One. 2023 Mar 2;18(3):e0282515. doi: 10.1371/journal.pone.0282515 (PMC9980782; doi:10.1371/journal.pone.0282515)
Supplement: S3 Table — (DOCX) [file pone.0282515.s003.docx]

**S3 Table. Regression coefficients for intake of dry matter, β-carotene, ether extract and proportions of individual fatty acids of total consumed dietary lipids (average of 7 days) in relation to the percentage of grassland-based feed in the diet (%GB) and the year of harvest (n = 12, 2018; n = 12, 2019).**

|  | **Intercept** | **Explanatory variables** | | | | | | | **Model** | | |
| --- | --- | --- | --- | --- | --- | --- | --- | --- | --- | --- | --- |
| **Target variable** | **b_0_** | **%GB** | **%GBsq** | **Year** | **%GB × Year_2018_** | **%GB × Year_2019_** | **DIM** | **Adj. R^2^** | | ***P* value** |  |
| DMI (kg/d) | 18.1 | ‒0.0467 | ‒^1^ | ‒ | ‒ | ‒ | 0.0137^*^ | 0.376 | | 0.003 |  |
| β-carotene intake (mg/d)^2^ | 32.4 | 17.61 | ‒ | ‒ | ‒8.44 | 0 | 1.04 | 0.870 | | <0.001 |  |
| Ether extract intake (g/d) | 658 | ‒ | ‒0.0197^**^ | ‒ | ‒ | ‒ | 0.443^*^ | 0.471 | | 0.001 |  |
| Fatty acids (mg/100 g total FAME) | | | | | | | | | | | |
| C8:0 | 112 | ‒0.352 | ‒ | 28.4^**^ | ‒ | ‒ | 0.0745^*^ | 0.630 | | <0.001 |  |
| C10:0 | 156 | ‒0.496^*^ | ‒ | 23.3^**^ | ‒ | ‒ | 0.107^*^ | 0.512 | | 0.001 |  |
| C12:0 | 4581 | 71.7 | 0.438 | ‒ | ‒ | ‒ | 1.71^*^ | 0.391 | | 0.005 |  |
| C12:1 | 129 | 5.84 | ‒ | 138^**^ | ‒ | ‒ | 0.481^*^ | 0.692 | | <0.001 |  |
| C14:0 | 1456 | 0.507 | ‒ | 229^*^ | ‒ | ‒ | 1.32^*^ | 0.355 | | 0.008 |  |
| C15:0 | 394 | 0.629 | ‒ | 249^***^ | ‒ | ‒ | ‒ | 0.776 | | <0.001 |  |
| C16:0 | 91274 | ‒407^**^ | ‒ | 9877^*^ | ‒ | ‒ | 47.1 | 0.561 | | 0.001 |  |
| *iso*-C16:0 | 672 | 60.0^***^ | ‒ | ‒ | ‒ | ‒ | 4.49^*^ | 0.658 | | <0.001 |  |
| C16:1n-7 | 1258 | ‒ | ‒0.0448^**^ | ‒ | ‒ | ‒ | 0.607 | 0.483 | | 0.001 |  |
| *anteiso*-C16:0 | ‒412 | 2.55^**^ | ‒0.0204^**^ | ‒ | ‒ | ‒ | ‒ | 0.653 | | <0.001 |  |
| C17:0 | 867 | ‒4.83^***^ | ‒ | 310^***^ | ‒ | ‒ | 0.397 | 0.811 | | <0.001 |  |
| C18:0 | 22834 | ‒170^***^ | ‒ | 2381^***^ | ‒ | ‒ | ‒ | 0.816 | | <0.001 |  |
| *trans*-9 C18:1 | ‒123 | 7.54^**^ | ‒0.0602^**^ | ‒ | ‒ | ‒ | ‒ | 0.653 | | <0.001 |  |
| *trans*-10 C18:1 | ‒145 | 8.89^**^ | ‒0.0710^**^ | ‒ | ‒ | ‒ | ‒ | 0.653 | | <0.001 |  |
| *trans*-11 C18:1 (VA) | ‒128 | 7.84^**^ | ‒0.0626^**^ | ‒ | ‒ | ‒ | ‒ | 0.653 | | <0.001 |  |
| *trans*-12 C18:1 | ‒279 | 1.71^**^ | ‒0.0136^**^ | ‒ | ‒ | ‒ | ‒ | 0.653 | | <0.001 |  |
| *cis*-9 C18:1 | 193849 | ‒ | ‒17.5^***^ | ‒ | ‒ | ‒ | ‒ | 0.917 | | <0.001 |  |
| *cis*-11 C18:1 | 1093 | 167 | ‒1.54^*^ | ‒ | ‒ | ‒ | ‒ | 0.714 | | <0.001 |  |
| *cis*-13 C18:1 | ‒40.1 | 2.47^**^ | ‒0.0197^**^ | ‒ | ‒ | ‒ | ‒ | 0.653 | | <0.001 |  |
| C18:2n-6 (LA) | 243337 | ‒ | ‒20.6^***^ | ‒ | ‒ | ‒ | 96.5 | 0.889 | | <0.001 |  |
| C18:3n-6 (GLA) | 247 | 5.24^***^ | ‒ | ‒ | ‒1.58^**^ | 0 | 0.544^*^ | 0.530 | | 0.001 |  |
| C18:3n-3 (ALA) | 16291 | 2242^***^ | ‒ | - | ‒570^***^ | 0 | 146^*^ | 0.722 | | <0.001 |  |
| C20:0 | 4415 | ‒28.7^***^ | ‒ | 695^***^ | ‒ | ‒ | 1.81 | 0.765 | | <0.001 |  |
| C20:1n9 | 2697 | 0.579 | ‒ | ‒ | ‒20.3^***^ | 0 | 1.53 | 0.885 | | <0.001 |  |
| C20:1n7 | 251 | ‒1.09^**^ | ‒ | 23.4^*^ | ‒ | ‒ | 0.149^*^ | 0.567 | | 0.001 |  |
| C21:0 | 371 | ‒1.96^***^ | ‒ | 130^***^ | ‒ | ‒ | 0.173 | 0.794 | | <0.001 |  |
| C20:2n-6 | 286 | ‒0.0596 | ‒ | 51.6^*^ | ‒ | ‒ | 0.200 | 0.343 | | 0.010 |  |
| C22:0 | 2372 | 1.53 | ‒ | 818^***^ | ‒ | ‒ | 2.176 | 0.546 | | 0.001 |  |
| C20:3n-3 | 38.8 | 0.143 | ‒ | 37.3^***^ | ‒ | ‒ | ‒ | 0.842 | | <0.001 |  |
| C22:1 | 297 | 0.544 | ‒ | 111^***^ | ‒ | ‒ | 0.245 | 0.571 | | 0.001 |  |
| C20:5n-3 (EPA) | 193 | ‒0.771^*^ | ‒ | 98.7^***^ | ‒ | ‒ | 0.114 | 0.828 | | <0.001 |  |
| C23:0 | 839 | ‒3.86^**^ | ‒ | 484^***^ | ‒ | ‒ | ‒ | 0.871 | | <0.001 |  |
| C22:2 | 649 | ‒3.63^**^ | ‒ | 174^***^ | ‒ | ‒ | ‒ | 0.732 | | <0.001 |  |
| C24:0 | 3055 | ‒11.0^*^ | ‒ | 785^***^ | ‒ | ‒ | 1.87^*^ | 0.665 | | <0.001 |  |
| C24:1n9 | 258 | 0.585 | ‒ | ‒ | ‒1.51^***^ | 0 | 0.189^*^ | 0.721 | | <0.001 |  |
| Σ SFA | 128747 | ‒562 | ‒ | 15683^*^ | ‒ | ‒ | 67.6 | 0.566 | | 0.001 |  |
| Σ MUFA | 207567 | ‒ | ‒18.04 | ‒ | ‒ | ‒ | ‒ | 0.909 | | <0.001 |  |
| Σ PUFA | 355551 | ‒ | ‒8.79 | ‒ | ‒ | ‒ | 187 | 0.326 | | 0.006 |  |

ALA, α-linolenic acid; DIM, days in milk; DMI, dry matter intake; EPA, eicosapentaenoic acid; FAME, fatty acid methyl ester; GB, grassland based; GLA, γ-linolenic acid; LA, linoleic acid; MUFA, monounsaturated fatty acids; PUFA, polyunsaturated fatty acids;; SFA, saturated fatty acids; VA, vaccenic acid.

^1^Variable not included in the final regression model after the variable selection procedure.

^2^Excluding the intakes of the cows supplemented with β-carotene. ^***^*P* < 0.001, ^**^*P* < 0.01, ^*^*P* < 0.05.
