# Supplementary material for: Evaluation and quantification of associations between commonly suggested milk biomarkers and the proportion of grassland-based feeds in the diets of dairy cows
Source: PLoS One. 2023 Mar 2;18(3):e0282515. doi: 10.1371/journal.pone.0282515 (PMC9980782; doi:10.1371/journal.pone.0282515)
Supplement: S4 Table — (DOCX) [file pone.0282515.s004.docx]

**S4 Table. Individual milk fatty acids or groups of fatty acids (% of total FAME) proportions affected by the year of harvest of grassland-based feeds as analyzed by GC.**

|  | **Year of harvest** | |  |  |
| --- | --- | --- | --- | --- |
| **Item** | **2018** | **2019** | **Min ‒ Max** | ***P* value** |
| C4:0 | 1.39 ± 0.23 | 1.62 ± 0.23 | 0.90 ‒ 2.06 | 0.021 |
| C6:0 | 1.42 ± 0.18 | 1.76 ± 0.15 | 1.17 ‒ 1.97 | <0.001 |
| C10:0 | 2.28 ± 0.44 | 3.16 ± 0.50 | 1.52 ‒ 4.29 | <0.001 |
| C10:1 | 0.26 ± 0.06 | 0.34 ± 0.06 | 0.13 ‒ 0.44 | 0.004 |
| C12:0 | 2.61 ± 0.52 | 3.67 ± 0.64 | 1.60 ‒ 5.15 | <0.001 |
| *iso*-C12:0 | 0.07 ± 0.02 | 0.09 ± 0.02 | 0.03 ‒ 0.12 | 0.006 |
| C12:1 | 0.08 ± 0.02 | 0.11 ± 0.02 | 0.04 ‒ 0.14 | 0.003 |
| C13:0 | 0.09 ± 0.02 | 0.13 ± 0.02 | 0.06 ‒ 0.16 | <0.001 |
| C14:0 | 10.7 ± 1.7 | 12.4 ± 1.1 | 7.2 ‒ 14.2 | 0.007 |
| *anteiso*-C14:0 | 0.50 ± 0.09 | 0.57 ± 0.05 | 0.35 ‒ 0.68 | 0.020 |
| *iso*-C16:0 | 0.24 ± 0.04 | 0.21 ± 0.04 | 0.15 ‒ 0.32 | 0.078 |
| C16:1n-7 | 2.53 ± 0.46 | 1.79 ± 0.27 | 1.39 ‒ 3.14 | <0.001 |
| *anteiso*-C16:0 | 0.07 ± 0.03 | 0.04 ± 0.01 | 0.02 ‒ 0.15 | 0.003 |
| C17:0 | 0.64 ± 0.06 | 0.56 ± 0.06 | 0.47 ‒ 0.75 | 0.002 |
| *iso*-C17:0 | 0.06 ± 0.01 | 0.09 ± 0.02 | 0.03 ‒ 0.12 | 0.001 |
| C17:1 | 0.35 ± 0.07 | 0.23 ± 0.03 | 0.17 ‒ 0.50 | <0.001 |
| *anteiso*-C17:0 | 0.07 ± 0.02 | 0.06 ± 0.01 | 0.04 ‒ 0.10 | 0.060 |
| C18:1n-9 | 24.4 ± 4.1 | 20.0 ± 2.4 | 15.0 ‒ 31.2 | 0.004 |
| *cis*-10 C18:1 | 0.20 ± 0.03 | 0.29 ± 0.08 | 0.16 ‒ 0.47 | 0.001 |
| *cis-*11 C18:1 | 0.52 ± 0.17 | 0.32 ± 0.05 | 0.24 ‒ 0.92 | 0.001 |
| *cis*-13 C18:1 | 0.12 ± 0.04 | 0.07 ± 0.02 | 0.06 ‒ 0.23 | 0.007 |
| *cis*-14 + *trans*-16 C18:1 | 0.28 ± 0.04 | 0.43 ± 0.12 | 0.22 ‒ 0.70 | 0.001 |
| *trans*-12 C18:1 | 0.19 ± 0.09 | 0.27 ± 0.12 | 0.05 ‒ 0.47 | 0.056 |
| C18:2n-6 (LA) | 1.33 ± 0.18 | 1.08 ± 0.16 | 0.88 ‒ 1.63 | 0.002 |
| *cis*-9, *cis*-15 C18:2 | 0.04 ± 0.02 | 0.03 ± 0.01 | 0.02 ‒ 0.07 | 0.036 |
| *cis*-9, *trans*-12 C18:2 | 0.10 ± 0.01 | 0.11 ± 0.03 | 0.08 ‒ 0.17 | 0.072 |
| *trans*-6 C18:2 | 0.08 ± 0.02 | 0.10 ± 0.03 | 0.05 ‒ 0.16 | 0.036 |
| *cis*-5 C20:1 | 0.02 ± 0.003 | 0.02 ± 0.005 | 0.01 ‒ 0.03 | 0.015 |
| *trans*-C20:1 | 0.02 ± 0.003 | 0.02 ± 0.005 | 0.01 ‒ 0.04 | 0.093 |
| C22:5n-3 | 0.06 ± 0.01 | 0.07 ± 0.01 | 0.04 ‒ 0.09 | 0.057 |
| C22:6n-3 | 0.008 ± 0.002 | 0.01 ± 0.003 | 0.005 ‒ 0.02 | 0.002 |
| Σ SFA | 61.4 ± 4.3 | 66.7 ± 3.4 | 53.3 ‒ 72.6 | 0.003 |
| Σ MUFA | 33.2 ± 4.3 | 28.1 ± 2.8 | 23.1 ‒ 40.9 | 0.002 |
| Σ n-6 | 1.80 ± 0.19 | 1.59 ± 0.23 | 1.33 ‒ 2.12 | 0.027 |

Arithmetic means ± SD, overall lowest and highest values, n = 12, 2018; n = 12, 2019), *P* values = one-way ANOVA with Year (categorical) as explanatory variable.

ALA, α-linolenic acid; EPA, eicosapentaenoic acid; GLA, γ-linolenic acid; LA, linoleic acid; MUFA, monounsaturated fatty acids; PUFA, polyunsaturated fatty acids; RA, rumenic acid; SFA, saturated fatty acids; VA, vaccenic acid.
