# Supplementary material for: Evaluation and quantification of associations between commonly suggested milk biomarkers and the proportion of grassland-based feeds in the diets of dairy cows
Source: PLoS One. 2023 Mar 2;18(3):e0282515. doi: 10.1371/journal.pone.0282515 (PMC9980782; doi:10.1371/journal.pone.0282515)
Supplement: S5 Table — (DOCX) [file pone.0282515.s005.docx]

|  | **Intercept** | **Explanatory variables** | | | | | | **Model** | |
| --- | --- | --- | --- | --- | --- | --- | --- | --- | --- |
| **Target variable** | **b_0_** | **%GB** | **%GBsq** | **Year** | **%GB × Year_2018_** | **%GB × Year_2019_** | **DIM** | **Adj. R^2^** | ***P* value** |
| C8:0 | 96.3 | ‒0.0891 | ‒ | ‒ | ‒ | ‒ | ‒ | ‒0.038 | 0.631 |
| *iso*-C12:0 | 98.4 | ‒0.089 | ‒ | ‒21.3^*^ | ‒ | ‒ | ‒ | 0.188 | 0.053 |
| *iso*-C13:0 | 155 | ‒0.521 | ‒ | ‒ | ‒ | ‒ | 0.129 | 0.176 | 0.062 |
| *iso*-C14:0 | 332 | ‒0.528 | ‒ | ‒ | ‒ | ‒ | ‒ | ‒0.023 | 0.471 |
| C14:1 | 1207 | ‒0.917 | ‒ | ‒ | ‒ | ‒ | ‒ | ‒0.046 | 0.775 |
| *iso*-C15:0 | 26.1 | ‒ | 0.00107 | ‒ | ‒ | ‒ | ‒ | 0.077 | 0.113 |
| C16:0 | 32813 | ‒29.9 | ‒ | ‒ | ‒ | ‒ | ‒ | ‒0.028 | 0.520 |
| C16:1 | 584 | ‒0.337 | ‒ | ‒ | ‒ | ‒ | ‒ | ‒0.031 | 0.550 |
| C18:0 | 13477 | ‒36.9 | ‒ | ‒ | ‒ | ‒ | ‒ | 0.048 | 0.167 |
| *trans*-9, C18:1 | 199 | ‒0.397 | ‒ | ‒ | ‒ | ‒ | ‒ | 0.012 | 0.277 |
| *trans*-10, C18:1 | 325 | ‒0.517 | ‒ | ‒ | ‒ | ‒ | ‒ | ‒0.042 | 0.702 |
| *trans*-11, C18:1 (VA) | 664 | 5.72 | ‒ | ‒ | ‒ | ‒ | ‒ | 0.113 | 0.070 |
| *trans*-12, C18:1 | 358 | ‒1.09 | ‒ | ‒94.4 | ‒ | ‒ | ‒ | 0.130 | 0.103 |
| *cis*-9, *cis*-11 C18:2 | 3.41 | 0.0330 | ‒ | ‒ | ‒ | ‒ | ‒ | ‒0.026 | 0.500 |
| *trans*-9, *trans*-11 C18:2 | ‒81.8 | 2.81^*^ | ‒0.0182 | ‒ | ‒ | ‒ | ‒ | 0.116 | 0.120 |
| C18:3n-6 (GLA) | 29.4 | 0.0137 | ‒ | ‒ | ‒ | ‒ | 0.0219 | 0.045 | 0.251 |
| *cis*-9 C20:1 | 37.9 | 0.120 | ‒ | ‒ | ‒ | ‒ | ‒ | ‒0.001 | 0.334 |
| *trans* C20:1 | ‒22.6 | 1.25 | ‒0.00855 | ‒ | ‒ | ‒ | ‒ | 0.107 | 0.132 |
| C20:2n-6 | ‒32.4 | 0.0146 | ‒00945 | ‒ | ‒ | ‒ | ‒ | 0.087 | 0.163 |
| C20:3n-6 | 81.7 | ‒0.154 | ‒ | ‒ | ‒ | ‒ | ‒ | ‒0.028 | 0.522 |
| C20:4n-6 | 98.3 | 0.00676 | ‒ | ‒ | ‒ | ‒ | ‒ | ‒0.050 | 0.979 |
| C20:3n-3 | 15.6 | 0.0160 | ‒ | ‒ | ‒ | ‒ | ‒ | ‒0.047 | 0.800 |
| C20:4n-3 | 12.8 | ‒0.0472 | ‒ | ‒ | ‒ | ‒ | ‒ | 0.030 | 0.214 |
| C20:5n-3 (EPA) | 52.0 | ‒0.0852 | ‒ | ‒ | ‒ | ‒ | ‒ | ‒0.034 | 0.585 |
| C22:0 | 78.5 | ‒0.268 | ‒ | ‒ | ‒ | ‒ | ‒ | 0.118 | 0.065 |
| C22:1 | 42.2 | 0.0243 | ‒ | ‒ | ‒ | ‒ | ‒ | ‒0.050 | 0.921 |
| C22:4n-6 | 53.4 | ‒0.206 | ‒ | ‒ | ‒ | ‒ | ‒ | 0.027 | 0.222 |
| C22:5n-3 | 77.9 | ‒0.134 | ‒ | ‒11.6^*^ | ‒ | ‒ | ‒ | 0.183 | 0.057 |
| C22:5n-6 | 11.4 | ‒0.0232 | ‒ | ‒ | ‒0.020 | 0 | ‒ | 0.122 | 0.112 |
| Σ n-6 | 1522 | 0.903 | ‒ | ‒ | 2.672^*^ | 0 | ‒ | 0.151 | 0.081 |

**S5. Table. Insignificant model regression coefficients for proportions of individual fatty acids (mg/100g total FAME) in milk analyzed by GC in relation to the percentage of grassland-based feeds in the diet (%GB) and the year of harvest (n=11, 2018; n=11, 2019).**

DIM, days in milk; EPA, eicosapentaenoic acid; GB, grassland based; GLA, γ-linolenic acid.

^1^Variable not included in the final regression model after the variable selection procedure.

^***^*P* < 0.001, ^**^*P* < 0.01, ^*^*P* < 0.05.
